# Recruitability and effect of PEEP in SARS-Cov-2-associated acute respiratory distress syndrome

**DOI:** 10.1186/s13613-020-00675-7

**Published:** 2020-05-12

**Authors:** François M. Beloncle, Bertrand Pavlovsky, Christophe Desprez, Nicolas Fage, Pierre-Yves Olivier, Pierre Asfar, Jean-Christophe Richard, Alain Mercat

**Affiliations:** 1 Département de Médecine Intensive-Réanimation, CHU d’Angers, Université d’Angers, 4 rue Larrey, 49933 Angers Cedex 9, France; 2 INSERM, UMR 955, Créteil, France

**Keywords:** Covid-19, SARS-Cov-2, Acute respiratory distress syndrome, Respiratory failure, Mechanical ventilation, Respiratory mechanics, Recruitability, Positive end-expiratory pressure

## Abstract

**Background:**

A large proportion of patients with a SARS-Cov-2-associated respiratory failure develop an acute respiratory distress syndrome (ARDS). It has been recently suggested that SARS-Cov-2-associated ARDS may differ from usual non-SARS-Cov-2-associated ARDS by higher respiratory system compliance (*C*_RS_), lower potential for recruitment with positive end-expiratory pressure (PEEP) contrasting with severe shunt fraction. The purpose of the study was to systematically assess respiratory mechanics and recruitability in SARS-Cov-2-associated ARDS.

**Methods:**

Gas exchanges, *C*_RS_ and hemodynamics were assessed at 2 levels of PEEP (15 cmH_2_O and 5 cmH_2_O) within 36 h (day1) and from 4 to 6 days (day 5) after intubation. The recruited volume was computed as the difference between the volume expired from PEEP 15 to 5 cmH_2_O and the volume predicted by compliance at PEEP 5 cmH_2_O (or above airway opening pressure). The recruitment-to-inflation (R/I) ratio (i.e. the ratio between the recruited lung compliance and *C*_RS_ at PEEP 5 cmH_2_O) was used to assess lung recruitability. A R/I ratio value higher than or equal to 0.5 was used to define highly recruitable patients.

**Results:**

The R/I ratio was calculated in 25 of the 26 enrolled patients at day 1 and in 15 patients at day 5. At day 1, 16 (64%) were considered as highly recruitable (R/I ratio median [interquartile range] 0.7 [0.55–0.94]) and 9 (36%) were considered as poorly recruitable (R/I ratio 0.41 [0.31–0.48]). The PaO_2_/FiO_2_ ratio at PEEP 15 cmH_2_O was higher compared to PEEP 5 cmH_2_O only in highly recruitable patients (173 [139–236] vs 135 [89–167] mmHg; *p* < 0.01). Neither PaO_2_/FiO_2_ or *C*_RS_ measured at PEEP 15 cmH_2_O or at PEEP 5 cmH_2_O nor changes in PaO_2_/FiO_2_ or *C*_RS_ in response to PEEP changes allowed to identify highly or poorly recruitable patients.

**Conclusion:**

In this series of 25 patients with SARS-Cov-2 associated ARDS, 64% were considered as highly recruitable and only 36% as poorly recruitable based on the R/I ratio performed on the day of intubation. This observation suggests that a systematic R/I ratio assessment may help to guide initial PEEP titration to limit harmful effect of unnecessary high PEEP in the context of Covid-19 crisis.

## Introduction

A very large proportion of patients admitted to ICU for Coronavirus disease 2019 (Covid-19) fulfill acute respiratory distress syndrome (ARDS) criteria according to Berlin definition [1–3]. In a large series of Severe Acute Respiratory Syndrome Coronavirus 2 (SARS-Cov-2)-associated respiratory failure, the majority of intubated patients were ventilated with high level of positive end-expiratory pressure (PEEP) [1]. However, information about specific individual characteristics of respiratory mechanics of SARS-Cov-2-associated ARDS remains very limited [4–6]. Based on their experience during the crisis, some authors suggested that part of the SARS-Cov-2 associated ARDS may present relatively high respiratory system compliance (*C*_RS_) and poor recruitability with PEEP, contrasting with severe hypoxemia. Accordingly, high levels of PEEP might be harmful in this so-called “phenotype”. The aim of this prospective study is to describe the characteristics of the respiratory mechanics of SARS-Cov-2-associated ARDS, and, in particular, whether the lungs are recruitable with high levels of PEEP.

## Materials and methods

### Study design and patient selection

Patients admitted from March 18th 2020 to April 2nd 2020 to the medical ICU of the university hospital of Angers and intubated for SARS-Cov-2-associated ARDS were prospectively included within 24 h of intubation. ARDS was defined according to the Berlin definition criteria [3]. SARS-Cov-2 infection was confirmed by real-time reverse transcriptase-polymerase chain reaction (RT-PCR) assay of nasal swabs or lower respiratory tract samples (bronchoalveolar lavage or endotracheal aspirate). Exclusion criteria were age lower than 18 years, pneumothorax and use of extracorporeal membrane oxygenation (ECMO).

### Study protocol

#### Ventilation and sedation strategy

Our care strategy did not include high-flow nasal canula (HFNC), continuous positive airway pressure (CPAP) or non-invasive ventilation for the management of patients with Covid-19.

After intubation, patients received initially deep sedation and neuromuscular blockers for 24 to 48 h and were ventilated in volume-controlled mode with a tidal volume of 6 mL/kg of predicted body weight (PBW) and a respiratory rate up to 35/min, adjusted to maintain arterial pH above 7.30. PEEP was set according to gas exchange, hemodynamic tolerance and a plateau pressure lower or equal to 28 cmH_2_O. The fraction of inspired oxygen (FiO_2_) was set for an arterial oxygen saturation (SaO_2_) between 92 and 98%.

#### Assessment of recruitment and detection of airway closure

Lung recruitment induced by high PEEP and detection of airway closure were assessed as previously described [7, 8].

All the measurements were performed in supine semi-recumbent position, with the head of the bed elevated at 30°, in volume-controlled mode with tidal volume of 6 mL/kg PBW and a constant inspiratory flow of 60 L/min.

After 15 min at a PEEP level of 15 cmH_2_O, the respiratory rate was decreased to 10/min to eliminate possible intrinsic PEEP, and the expired tidal volume displayed by the ventilator was noted. PEEP was abruptly decreased to 5 cmH_2_O and expired volume displayed by the ventilator immediately after the maneuver was noted. The previous respiratory rate was resumed and PEEP was maintained at 5 cmH_2_O for the next 15 min.

Plateau pressure, total PEEP, arterial blood gases and central venous blood gases (collected from a jugular venous line) were assessed at the two levels of PEEP (Additional file 1: Figure S1). Mean arterial pressure (MAP) and heart rate were also recorded at the end of the application of each PEEP level.

A low-flow (5 L/min) inflation from PEEP 5 cmH_2_O (tidal volume = 9 mL/kg PBW) was then performed to identify a possible airway closure [8]. Airway closure was identified by the inspection of the pressure–time curve and the airway opening pressure (AOP) was measured using cursors on the ventilator screen.

This maneuver was performed in the supine position within 36 h after intubation (day 1) and from day 4 to day 6 after intubation (day 5) in patients still ventilated in volume-controlled mode, neither triggering the ventilator nor on ECMO.

The recruited lung volume was computed as the volume expired from PEEP 15 to 5 cmH_2_O (displayed on the ventilator screen immediately after an abrupt decrease in PEEP) subtracting from the previous expired tidal volume and from the lung volume predicted by the compliance at low PEEP (Additional file 1: Figure S1) [9, 10]. The lung volume predicted by the compliance at low PEEP represents the minimum predicted change in lung volume corresponding to the change in pressure between the 2 PEEP levels (i.e. the change in lung volume if no recruitment occurs) and is equal to the product of *C*_RS_ at PEEP 5 cmH_2_O (or above AOP) and PEEP change (i.e. 10 cmH_2_O or 15-AOP).

The R/I ratio represents the ratio between the compliance of the recruited lung and the compliance of the “baby lung”. Briefly, the compliance of the recruited lung was calculated as the recruited lung volume divided by the difference between the 2 PEEP levels (i.e. 10 cmH_2_O) in patients without airway closure at 5 cmH_2_O or by the difference between 15 cmH_2_O and AOP, in patients with airway closure above 5 cmH_2_O [7]. The respiratory system compliance at PEEP 5 cmH_2_O or above AOP was used as a surrogate for the compliance of the baby lung. A high R/I ratio is considered to be associated with a high potential for lung recruitment. As previously described, a threshold of 0.5 was used to differentiate poorly recruitable from highly recruitable patients [7].

### Other collected and measured data

The following data were collected at inclusion (i.e. on the day of intubation): age, past medical history, Sequential Organ Failure Assessment (SOFA) score [11] and simplified acute physiologic score II (SAPS II) [12], partial pressure of arterial oxygen (PaO_2_), fraction of inspired oxygen (FiO_2_), partial pressure of arterial carbon dioxide (PaCO_2_), tidal volume, respiratory rate, minute ventilation, set PEEP and plateau pressure. The delay from symptom onset to ICU admission and from ICU admission to intubation was also reported.

The estimated shunt fraction was calculated, based on the venous admixture determination [13], considering central venous oxygen saturation (ScVO_2_) as an acceptable surrogate for mixed venous oxygen saturation [14]:

$${\text{Estimated shunt fraction: }}{{Q_{VA} } \mathord{\left/ {\vphantom {{Q_{VA} } {Q_{T} \left( \% \right)}}} \right. \kern-0pt} {Q_{T} \left( \% \right)}}\, \approx \,{{\left( {{\text{CcO}}_{2} {-}{\text{CaO}}_{2} } \right)} \mathord{\left/ {\vphantom {{\left( {{\text{CcO}}_{2} {-}{\text{CaO}}_{2} } \right)} {\left( {{\text{CcO}}_{2} {-}{\text{CvO}}_{2} } \right)}}} \right. \kern-0pt} {\left( {{\text{CcO}}_{2} {-}{\text{CvO}}_{2} } \right),}}$$

with CaO_2_, CvO_2_ and CcO_2_ being the arterial, central venous, and ideal capillary O_2_ concentration values, respectively.

The *C*_RS_ was computed as tidal volume divided by the difference between plateau pressure and total PEEP (or AOP in patients with airway closure at 5 cmH_2_O).

For each patient, the extension and severity of lung opacities were assessed on the first chest X-ray performed after the intubation by 2 independent observers who were unaware of the patient’s clinical data, using the RALE score [15]. In this score, each quadrant is scored for extent of consolidation (from 0 to 4) and density of opacification (from 1 to 3). The RALE score corresponds to the sum of the products of the consolidation and density scores of each of the 4 quadrants (maximum score = 48).

### Statistical analysis

Data are presented as median [interquartile range] or number (percentage). The study population was divided into 2 groups according to the R/I ratio at day 1. Highly recruitable patients group was composed of patients with R/I ratio higher than or equal to 0.5 at day 1 and the patients with R/I ratio lower than 0.5 made up the poorly recruitable patients group. The two groups of patients were compared using Mann–Whitney U-test or Fisher’s exact test as appropriate. Paired data were compared using Wilcoxon test for paired data. All tests were performed with a type I error set at 0.05. The statistical analysis was performed using Prism (GraphPad Software v5.0b, La Jolla, CA, USA).

## Results

### Patients characteristics

Twenty-six patients were included in this study. The PEEP trial maneuver for recruitability assessment was rapidly interrupted in one patient at day 1 because of major desaturation on PEEP 5 cmH_2_O. Twenty-five patients were thus analyzed.

Main characteristics of the patients and respiratory parameters on the day of intubation are presented in Tables 1 and 2.

**Table 1:** Baseline characteristics of study patients

	All patients, *n* = 25	Highly recruitable, *n* = 16	Poorly recruitable, *n* = 9	*p* value
Age, years	71 [60.5–78]	71.5 [63–76]	67 [54–75.5]	0.43
Male sex, n (%)	18 (72)	11 (69)	7 (78)	1
Height, cm	173 [165–179]	172 [165–180]	175 [172–177]	0.92
BMI, kg/m^2^	29.1 [25–32.3]	29 [24.8–32.6]	29.1 [25.3–32]	0.84
SOFA at enrollment	5 [3–7.5]	5.5 [3–8]	5 [3.5–6.5]	0.85
SAPS II at enrollment	44 [36–50]	44 [35.5–49.5]	44[36–51]	0.67
Pre-existing conditions, n (%)				
Hypertension	21 (84)	14 (88)	7 (77)	0.6
Diabetes mellitus	10 (40)	6 (37)	4 (44)	1
COPD/asthma	5 (20)	4 (25)	1 (11)	0.62
Smoking history	13 (52)	9 (56)	4 (44)	0.69
Delay from symptom onset to ICU admission, days	9 [7–12.5]	8 [7–10]	14 [7–15.5]	0.15
Delay from ICU admission to intubation, hours	0 [0–9]	0 [0–5.8]	9 [0–11]	0.28
Non-invasive support before intubation, n (%)	0 (0)	0 (0)	0 (0)	1

Data are presented as median [interquartile range] or number (percentage)

*BMI* body mass index, *SOFA* Sequential Organ Failure Assessment, *SAPS II* Simplified Acute Physiology Score II, *COPD* chronic obstructive pulmonary disease, *ICU* intensive care unit, Non-invasive support: high-flow nasal canula, continuous positive airway pressure or non-invasive ventilation

*p*-values refer to the comparison between the *highly* and *poorly recruitable patients* groups

**Table 2:** Respiratory parameters at inclusion

	All patients, *n* = 25	Highly recruitable, *n* = 16	Poorly recruitable, *n* = 9	*p* value
VT (ml/kg PBW)	6.0 [5.9–6.1]	6.1 [5.9–6.1]	6 [6–6.3]	0.59
RR/min	28 [26–30]	27.5 [25–30]	30 [28–31]	0.1
VE, L/min	12.3 [9.2–13]	11.9 [9.6–13.6]	12.6 [10.5–14.1]	0.85
FiO_2_, %	60 [40–65]	55 [40–70]	60 [45–65]	0.47
PEEP, cmH_2_O	12 [10–15]	13 [12–15]	10 [10–12]	0.02
Pplat, cmH_2_O	23 [21–24]	23 [20–24]	23 [21–27]	0.32
PaO_2_, mmHg	75 [65–94]	77 [68–90]	73 [62–99]	0.9
PaO_2_/FiO_2_, mmHg	135 [119–195]	140 [123–196]	121 [106–155]	0.43
PaCO_2_, mmHg	41 [38–44]	41 [38–42]	40 [36–45]	0.97
Patients with airway closure > 5 cmH_2_O, n (%)	6 (24)	4 (25)	2 (22)	1
AOP in patients with airway closure > 5 cmH_2_O, cmH_2_O	8 [7–10]	8 [7–10]	8 [6–10]	1

Data are presented as median [interquartile range] or number (percentage)

*VT* tidal volume, *PBW* predicted body weight, *RR* respiratory rate, *VE* minute ventilation, *FiO*_*2*_ fraction of inspired oxygen, *PEEP* set positive end-expiratory pressure, *Pplat* plateau pressure, *C*_RS_ compliance of the respiratory system, *PaO*_*2*_ partial pressure of arterial oxygen, *PaCO*_*2*_ partial pressure of arterial carbon dioxide, *AOP* airway opening pressure, *R/I ratio* recruitment-to-inflation ratio

*p*-values refer to the comparison between the *highly* and *poorly recruitable patients* groups

### Recruitment/inflation ratio assessments

The R/I ratio was assessed in 25 patients at day 1 and in 15 patients at day 5.

**Fig. 1:**
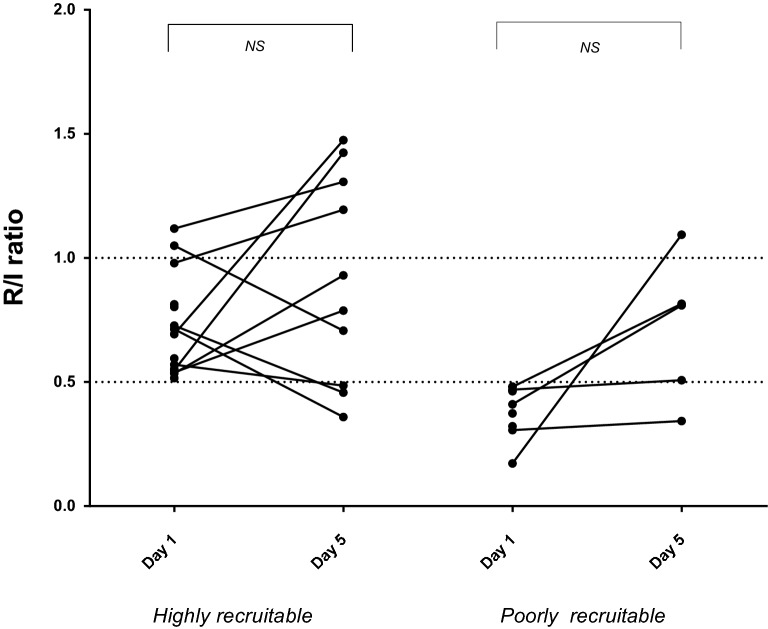
Distribution of recruitment/inflation ratio (R/I ratio) within 36 hours after intubation (Day 1) and from 4 to 6 days after intubation (Day 5) in the *highly recruitable* and *poorly recruitable patients* groups. *NS*, not significant (*p* > *0.05*)

Among the 25 patients evaluated at day 1, 16 (64%) were considered as highly recruitable (R/I ratio 0.70 [0.55–0.94]) and 9 (36%) were considered as poorly recruitable (R/I ratio 0.41 [0.31–0.48]); Table 3 and Fig. 1. The recruited lung volume on PEEP 15 cmH_2_O compared to PEEP 5 cmH_2_O was significantly higher in the highly recruitable patients than in the poorly recruitable patients (338 [245–454] mL vs 206 [91–275] mL; *p* < 0.01); Table 3 and Additional file 2: Figure S2.

**Table 3:** Recruitment/inflation ratio (R/I ratio), recruited lung volume (*V*_REC_) and respiratory system compliance (*C*_RS_) at positive end-expiratory pressure (PEEP) 5 cmH_2_O and 15 cmH_2_O, within 36 h after intubation in the highly recruitable and poorly recruitable patients groups

	All patients	Highly recruitable, *n* = 16	Poorly recruitable, *n* = 9	*p* value
R/I ratio	0.55 [0.47–0.77]	0.70 [0.55–0.94]	0.41 [0.31–0.48]	–
*V*_REC_, mL	277 [218–422]	338 [245–454]	206 [91–275]	< 0.01
*C*_RS_ at PEEP 5 cmH_2_O, mL/cmH_2_O	50 [38–64]	45 [38–66]	54 [33–63]	0.99
*C*_RS_ at PEEP 15 cmH_2_O, mL/cmH_2_O	45 [37–54]	45 [38–58]	45 [34–53]	0.67

Data are presented as median [interquartile range]

*p*-values refer to the comparison between the highly and poorly recruitable patients groups. R/I ratio is by definition higher in the highly recruitable than in the poorly recruitable patients

Among the 16 patients considered as highly recruitable at day 1, a second R/I ratio assessment was performed at day 5 in 10 patients. Among these 10 patients, 7 remained highly recruitable and 3 became poorly recruitable, Fig. 1. In addition, 1 patient was switched early to pressure support, 4 patients were discharged from ICU, and 1 died before the second R/I ratio assessment. Among the 9 patients considered as poorly recruitable at day 1, a second R/I ratio assessment was performed at day 5 in 5 patients. Among these 5 patients, 4 became highly recruitable and 1 remained poorly recruitable, Fig. 1. The 4 other patients were switched early to pressure support.

### Gas exchange, respiratory system compliance, hemodynamics and chest X-ray

**Fig. 2:**
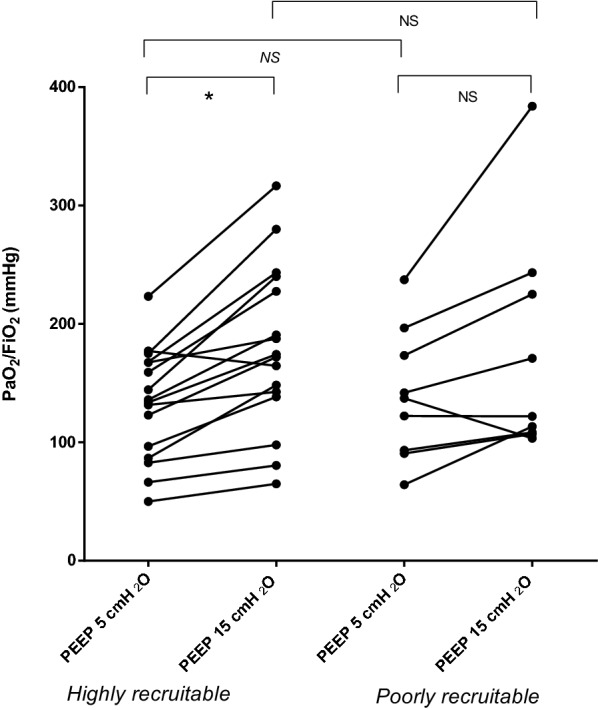
Distribution of ratio of partial pressure of arterial oxygen and fraction of inspired oxygen (PaO_2_/FiO_2_) at positive end-expiratory pressure (PEEP) 5 cmH_2_O and 15 cmH_2_O within 36 h after intubation in the *highly recruitable* and *poorly recruitable patients* groups. *, *p *<* 0.01*; *NS*, not significant (*p *> *0.05*)

The PaO_2_/FiO_2_ ratios measured at PEEP 5 cmH_2_O and at PEEP 15 cmH_2_O were not different in the 2 groups; Fig. 2. The PaO_2_/FiO_2_ ratio was higher at PEEP 15 cmH_2_O than at PEEP 5 cmH_2_O in the highly recruitable patients (173 [139–236] vs 135 [89–167] mmHg; *p* < 0.01), but not in the poorly recruitable patients (122 [108–234] mmHg at PEEP 15 cmH_2_O vs 137 [92–185] mmHg at PEEP 5 cmH_2_O; *p* = 0.06). Of note, compared to PEEP 5 cmH_2_O, PEEP 15 cmH_2_O was associated with an increase in the PaO_2_/FiO_2_ ratio of more than 20% in 12 patients (75%) in the highly recruitable patients group and in 5 patients (56%) in the poorly recruitable patients group (*p* = 0.39).

**Fig. 3:**
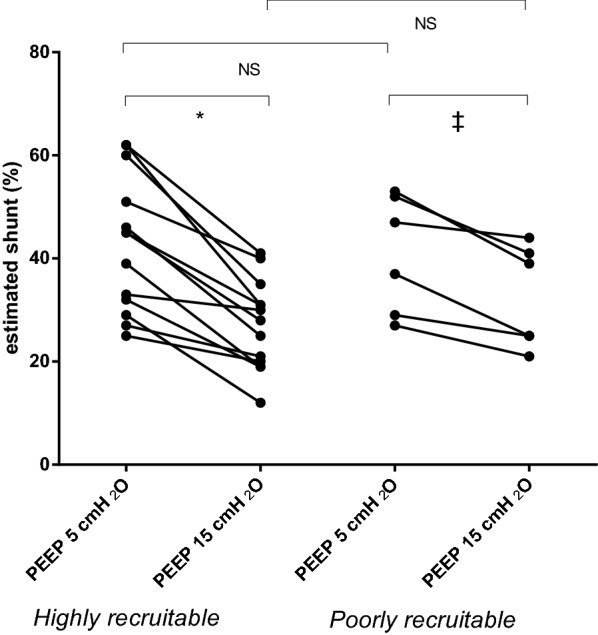
Distribution of estimated shunt at positive end-expiratory pressure (PEEP) 5 cmH_2_O and 15 cmH_2_O in the *highly recruitable* and *poorly recruitable patients* groups, within 36 h after intubation. Shunt was calculated with standard formula [13], using central venous blood gases as a surrogate for mixed venous blood gases [14]. *p <0.01. ^‡^*p *=0.03

The estimated shunt fraction (*n* = 19, 13 in highly recruitable patients, 6 in poorly recruitable patients) was lower at PEEP 15 cmH_2_O than at PEEP 5 cmH_2_O in both groups (28 [19.5–33] vs 45 [30.5–55.5] %, *p* < 0.01 in highly recruitable patients; 32 [24–41.8] vs 42 [28.5–52.3] %, *p* = 0.03 in poorly recruitable patients); Fig. 3.

**Fig. 4:**
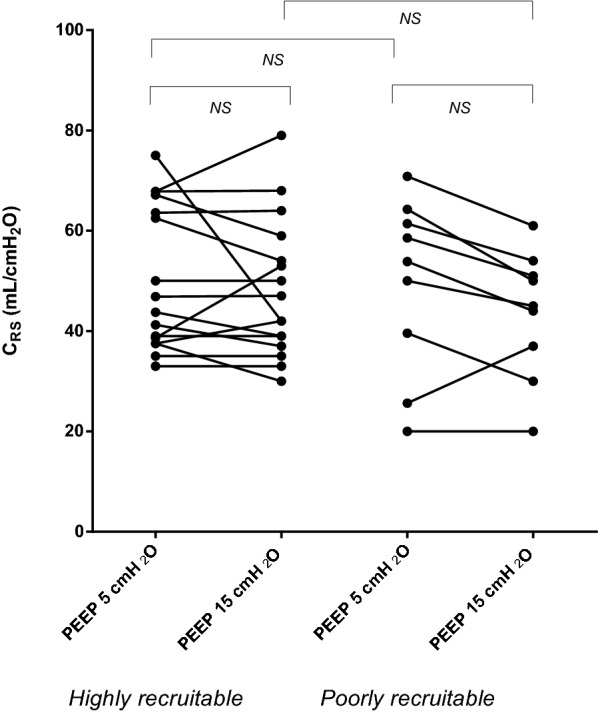
Distribution of respiratory system compliance (*C*_RS_) at positive end-expiratory pressure (PEEP) 5 cmH_2_O and 15 cmH_2_O in the highly recruitable and poorly recruitable patients groups, within 36 h after intubation. *NS*, not significant (*p *> 0.05)

The *C*_RS_ at PEEP 5 cmH_2_O or 15 cmH_2_O was not different in the 2 groups (45 [38–66] mL/cmH_2_O in the highly recruitable patients group vs 54 [33–63] mL/cmH_2_O in the poorly recruitable patients group at PEEP 5 cmH_2_O, *p* = 0.99 and 45 [38–58] mL/cmH_2_O in the highly recruitable patients group vs 45 [34–53] mL/cmH_2_O in the poorly recruitable patients group at PEEP 15 cmH_2_O, *p* = 0.67); Table 3 and Fig. 4. In addition, *C*_RS_ was not significantly altered by PEEP change from 15 to 5 cmH_2_O in each of the 2 groups; Fig. 4 and Additional file 3: Figure S3.

We did not observe significant changes in MAP and HR between PEEP 15 cmH_2_O and PEEP 5 cmH_2_O in the 2 groups; Additional file 4: Figure S4.

The RALE score of the chest X-ray performed after intubation did not differ between the 2 groups (14.5 [11–21] in the highly recruitable patients group vs 19 [14.5–33], in the poorly recruitable patients group, *p* = 0.2); Additional file 5: Figure S5.

## Discussion

The main results of the present case series could be summarized as follows: (1) the majority of these SARS-Cov-2-associated ARDS exhibit relatively preserved static *C*_RS_ and are considered as potentially recruitable based on R/I ratio soon after intubation; (2) neither individual values of PaO_2_/FiO_2_ ratio and static *C*_RS_ measured either at high or low PEEP nor changes of these parameters with change of PEEP allow to identify highly recruitable or poorly recruitable patients; (3) among patients initially considered as poorly recruitable, some of them become highly recruitable 5 days later.

In the present series of 25 SARS-Cov-2-associated ARDS, the initial respiratory mechanics assessment performed soon after intubation at PEEP 15 cmH_2_O and 5 cmH_2_O allowed to identify 16 patients as potentially highly recruitable and 9 as poorly recruitable based on the previously reported R/I ratio [7]. Interestingly, the second complete respiratory mechanics assessment performed 4 to 6 days later in 15 patients suggested that initial status may change more frequently from poorly recruitable to highly recruitable than the reverse.

The present small cases series is the first to describe early and complete respiratory mechanics evaluation in SARS-Cov-2-associated ARDS consistent with the “2 phenotypes” model proposed by Gattinoni et al. [16]. Our observations extend those initially reported in three small cases series showing essentially low or variable potentials of recruitment with PEEP [4–6].

In the recently reported Chinese series of 12 SARS-Cov-2-associated ARDS, all patients were initially considered as poorly recruitable based on the same method to measure R/I ratio [4]. Interestingly, repetitive measurements showed that some of them became recruitable potentially depending on the time course evolution of the disease and the respiratory treatment they received (notably prone positioning). Authors concluded that R/I ratio is feasible, even in the constrained Covid-19 environment and may allow to guide individual titration of PEEP to limit potential harmful effects expected with high PEEP in poorly recruitable patients. Most (75%) of these patients received NIV or HFNC before intubation for a median of 5 [IQR, 4–7] days. This could lead to patient self-inflicted lung injury [17] that may explain, at least in part, their low respiratory system compliance (around 20 mL/cmH_2_O) after intubation. This may also have participated to the apparent beneficial effect of prone position on recruitment in this series despite initial poor recruitability.

In the Italian series of 16 SARS-Cov-2-associated ARDS, the near-normal compliance of the respiratory system (50 ml/cmH_2_O on average) contrasted with severe hypoxemia suggesting relatively preserved lung volumes which is unusual in non-Covid-19 ARDS [5, 18]. Based on these original observations, authors challenged the classical recommendations for PEEP titration and prone positioning based on the severity of hypoxemia [19], suggesting that PEEP may lead to severe hemodynamic impairment and fluid retention in poorly recruitable patients while prone position may be less efficient imposing an unnecessary additional workload in the context of the pandemic. The same group of authors proposed a concise physiological description of what they called “phenotypes L and H”. Briefly they opposed the possible high proportion of poorly recruitable patients with near-normal compliance (“L”) to patients with low compliance and high potential for recruitment (“H” not different from classical non-Covid-19 ARDS) that may benefit from higher PEEP and prone position. Authors mentioned that the phenotype may change with time.

The mix of poorly recruitable and higly recruitable patients that we observed in the present study based on R/I ratio, roughly fit with this “H” and “L” description. In fact, PaO_2_/FiO_2_ ratio was significantly increased at PEEP 15 cmH_2_O compared to 5 cmH_2_O only in highly recruitable patients. Conversely, poorly recruitable patients exhibited a non-significant trend toward higher *C*_RS_ at low PEEP compared to highly recruitable patients. Moreover, the trend in decrease in *C*_RS_ observed in these patients when PEEP is increased may reflect overinflation thus indicating the risk associated with high PEEP in these patients. Of note, the increase in PaO_2_/FiO_2_ ratio with PEEP in poorly recruitable patients may be explained, at least in part, by a potential reduction in cardiac output induced by PEEP that may have contributed to decrease the shunt fraction [13]. Rather than the schematic opposition of two phenotypes, our results suggest that recruitment with PEEP in these patients must be individually evaluated since it may vary largely depending on the initial clinical presentation as well as the time course evolution under treatment.

Our study presents important limitations. First of all, the small number of patients enrolled in this series does not allow to conclude about the expected repartition of the two proposed phenotypes in a large population of SARS-Cov-2-associated ARDS. Moreover, initial respiratory management (i.e. prolonged use of HFNC, CPAP or non-invasive ventilation vs early intubation) is a confoundable parameter that may impact the respiratory pattern recorded immediately after intubation. Second, the respiratory mechanics characterization at two levels of PEEP and the R/I ratio do definitively not allow to determine accurately the optimal PEEP level. Finally, the second respiratory mechanics evaluation was not available in all patients thus limiting the possibility to assess the impact of prone positioning and PEEP settings that may change the evolution of the phenotype along the time course evolution of the disease.

## Conclusions

In this series of SARS-Cov2-associated ARDS, early respiratory mechanics assessment (at 15 and 5 cmH_2_O of PEEP) and R/I ratio calculation showed a mix of highly recruitable and poorly recruitable patients. Neither individual values of PaO_2_/FiO_2_ ratio or *C*_RS_ on low or high PEEP nor their changes after a change of PEEP allowed to distinguish highly recruitable from poorly recruitable patients. Present observations suggest that a systematic R/I ratio evaluation may be useful to guide initial setting of PEEP in the context of SARS-Cov2-associated ARDS.

## Supplementary information

**Additional file 1: Figure S1.** A. Study protocol. Positive end-expiratory pressure level (PEEP) was set to 15 cmH_2_O. Arterial and central venous blood gases were collected after a 10 min period and respiratory mechanics was assessed. Respiratory rate (RR) was decreased to 10/min and PEEP was decreased to 5 cmH_2_O (see below, Additional file 1: Figure S1B). After a 10 min period with PEEP 5 cmH_2_O, arterial and central venous blood gases were collected and respiratory mechanics was assessed. A low flow insufflation (5L/min) from PEEP 5 cmH_2_O was performed after a prolonged expiration. A visual analysis of the pressure–time curve on the ventilator screen allowed to identify a potential airway closure (and to measure a potential airway opening pressure) (see a representative tracing below). B. Measurement of the recruited lung volume. After decreasing RR to 10/min, expired tidal volume displayed by the ventilator at PEEP 15 cmH_2_O was noted. PEEP was abruptly decreased to 5 cmH_2_O and expired volume displayed by the ventilator immediately after the maneuver was noted. Plateau pressure at PEEP 5 cmH_2_O was measured. Initial RR was then resumed. C. Representative tracing of a low flow insufflation allowing to identify a complete airway closure and to measure the airway occlusion pressure (AOP).

**Additional file 2: Figure S2.** Distribution of recruited lung volume (*V*_REC_) within 36 h after intubation in the highly recruitable and poorly recruitable patients groups. *, *p* < 0.01. Horizontal lines represent median and interquartile range values.

**Additional file 3: Figure S3.** Distribution of changes in respiratory system compliance (∆*C*_RS_) from positive end-expiratory pressure (PEEP) 5 cmH_2_O to PEEP 15 cmH_2_O within 36 h after intubation in the highly recruitable and poorly recruitable patients groups. Horizontal lines represent median and interquartile range values. *NS*, *not significant* (*p* > *0.05*).

**Additional file 4: Figure S4.** Distribution of mean arterial pressure (MAP) (A) and heart rate (B) at positive end-expiratory pressure (PEEP) 5 cmH_2_O and 15 cmH_2_O in the highly recruitable and poorly recruitable patients groups. *NS*, *not significant* (*p* > *0.05*).

**Additional file 5: Figure S5.** Distribution of the *Radiographic Assessment of the quantity of Lung Edema* (RALE) score at the day of intubation in the highly recruitable and poorly recruitable groups. *NS*, *no significant* (*p* >* 0.05*). Horizontal lines represent median and interquartile range values.

## Data Availability

The datasets analyzed during the current study are available from the corresponding author on reasonable request

## Abbreviations

AOP: Airway opening pressure
ARDS: Acute respiratory distress syndrome
BMI: Body mass index
CaO_2_: Arterial oxygen concentration
CcO_2_: Ideal capillary oxygen concentration
CvO_2_: Central venous oxygen concentration
COPD: Chronic obstructive pulmonary disease
Covid-19: Coronavirus disease 2019
CPAP: Continuous positive airway pressure
*C*_RS_: Compliance of the respiratory system
ECMO: Extracorporeal membrane oxygenation
FiO_2_: Fraction of inspired oxygen
HFNC: High-flow nasal cannula
ICU: Intensive care unit
MAP: Mean arterial pressure
PaCO_2_: Partial pressure of arterial carbon dioxide
PaO_2_: Partial pressure of arterial oxygen
PBW: Predicted body weight
PEEP: Set positive end-expiratory pressure
Pplat: Plateau pressure
Q_VA_/Q_T_: Estimated shunt (venous admixture/total pulmonary blood flow)
R/I ratio: Recruitment-to-inflation ratio
RALE score: Radiographic assessment of the quantity of lung edema score
RR: Respiratory rate
RT-PCR: Reverse transcriptase-polymerase chain reaction
SaO_2_: Arterial oxygen saturation
SAPS II: Simplified Acute Physiology Score II
SARS-CoV-2: Severe Acute Respiratory Syndrome Coronavirus-2
SOFA: Sequential organ failure assessment
Vt: Tidal volume
