# Supplementary figures and images for: Recruitability and effect of PEEP in SARS-Cov-2-associated acute respiratory distress syndrome
Source: Ann Intensive Care. 2020 May 12;10:55. doi: 10.1186/s13613-020-00675-7 (PMC7215140; doi:10.1186/s13613-020-00675-7)

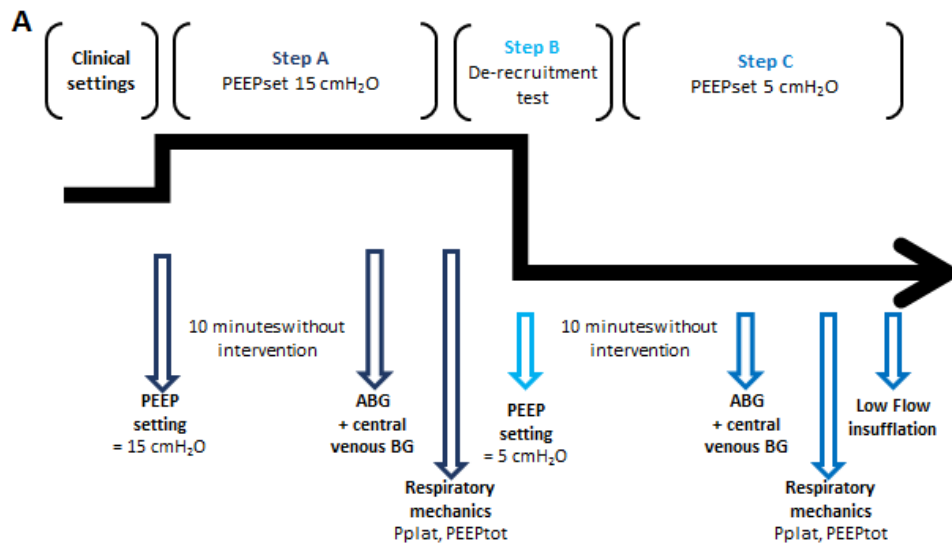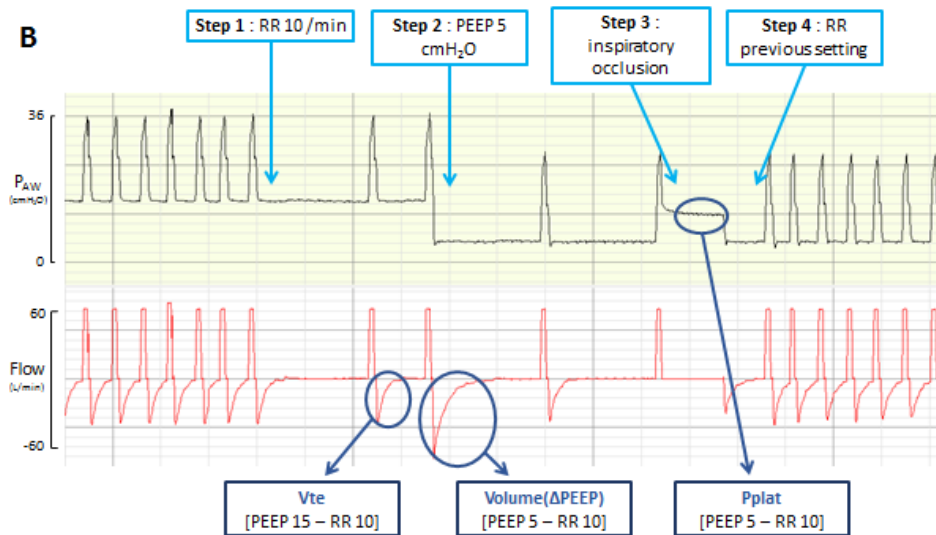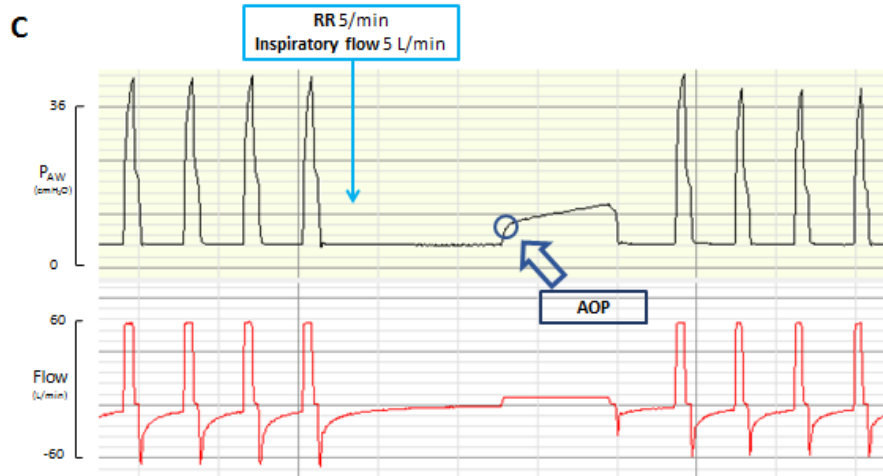

Supplement: Supplementary file 1 — Additional file 1: Figure S1. A. Study protocol. Positive end-expiratory pressure level (PEEP) was set to 15 cmH2O. Arterial and central venous blood gases were collected after a 10 min period and respiratory mechanics was assessed. Respiratory rate (RR) was decreased to 10/min and PEEP was decreased to 5 cmH2O (see below, Additional file 1: Figure S1B). After a 10 min period with PEEP 5 cmH2O, arterial and central venous blood gases were collected and respiratory mechanics was assessed. A low flow insufflation (5L/min) from PEEP 5 cmH2O was performed after a prolonged expiration. A visual analysis of the pressure–time curve on the ventilator screen allowed to identify a potential airway closure (and to measure a potential airway opening pressure) (see a representative tracing below). B. Measurement of the recruited lung volume. After decreasing RR to 10/min, expired tidal volume displayed by the ventilator at PEEP 15 cmH2O was noted. PEEP was abruptly decreased to 5 cmH2O and expired volume displayed by the ventilator immediately after the maneuver was noted. Plateau pressure at PEEP 5 cmH2O was measured. Initial RR was then resumed. C. Representative tracing of a low flow insufflation allowing to identify a complete airway closure and to measure the airway occlusion pressure (AOP). [file 13613_2020_675_MOESM1_ESM.pdf]

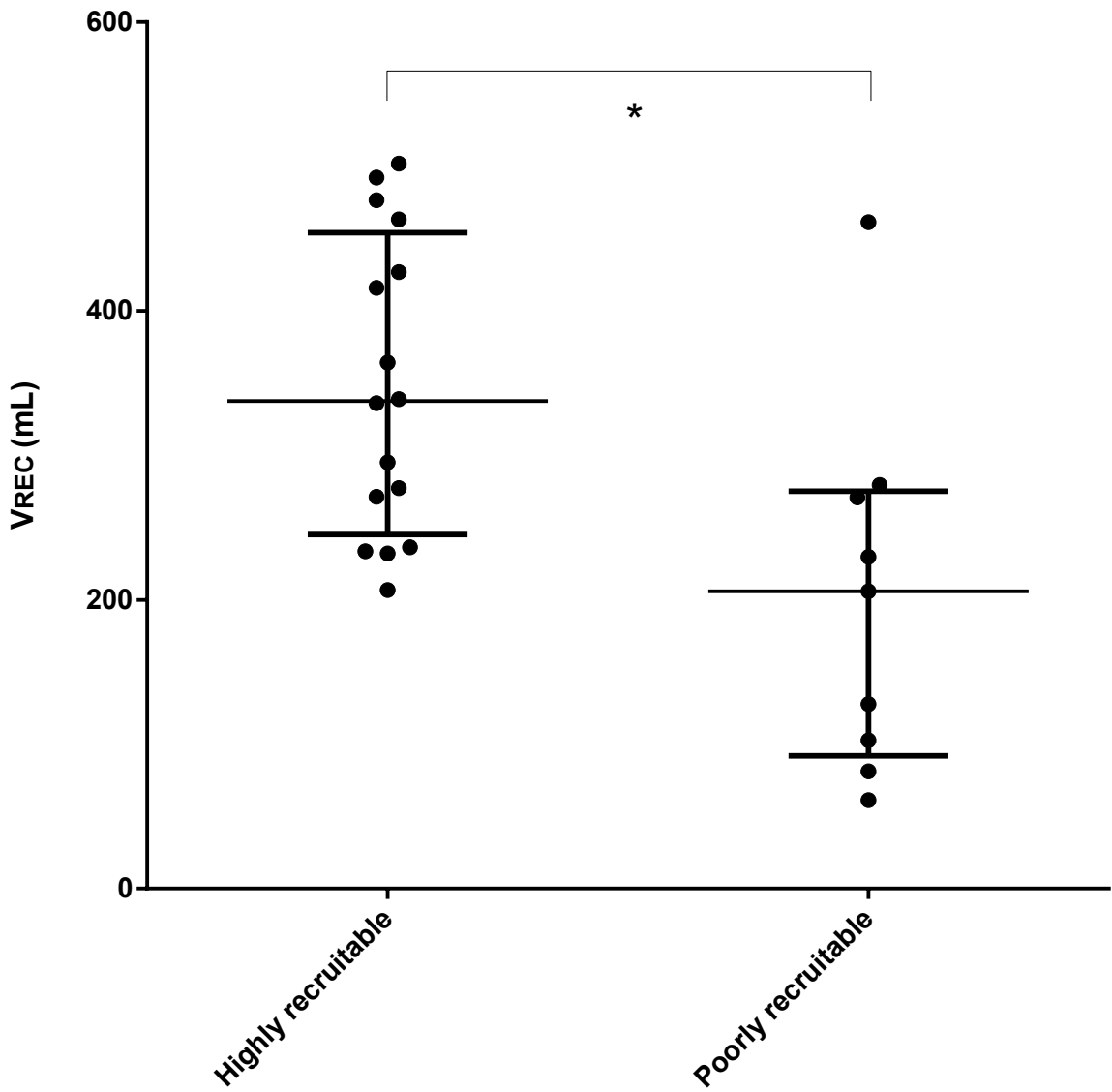

Supplement: Supplementary file 2 — Additional file 2: Figure S2. Distribution of recruited lung volume (VREC) within 36 h after intubation in the highly recruitable and poorly recruitable patients groups. *, p < 0.01. Horizontal lines represent median and interquartile range values. [file 13613_2020_675_MOESM2_ESM.pdf]

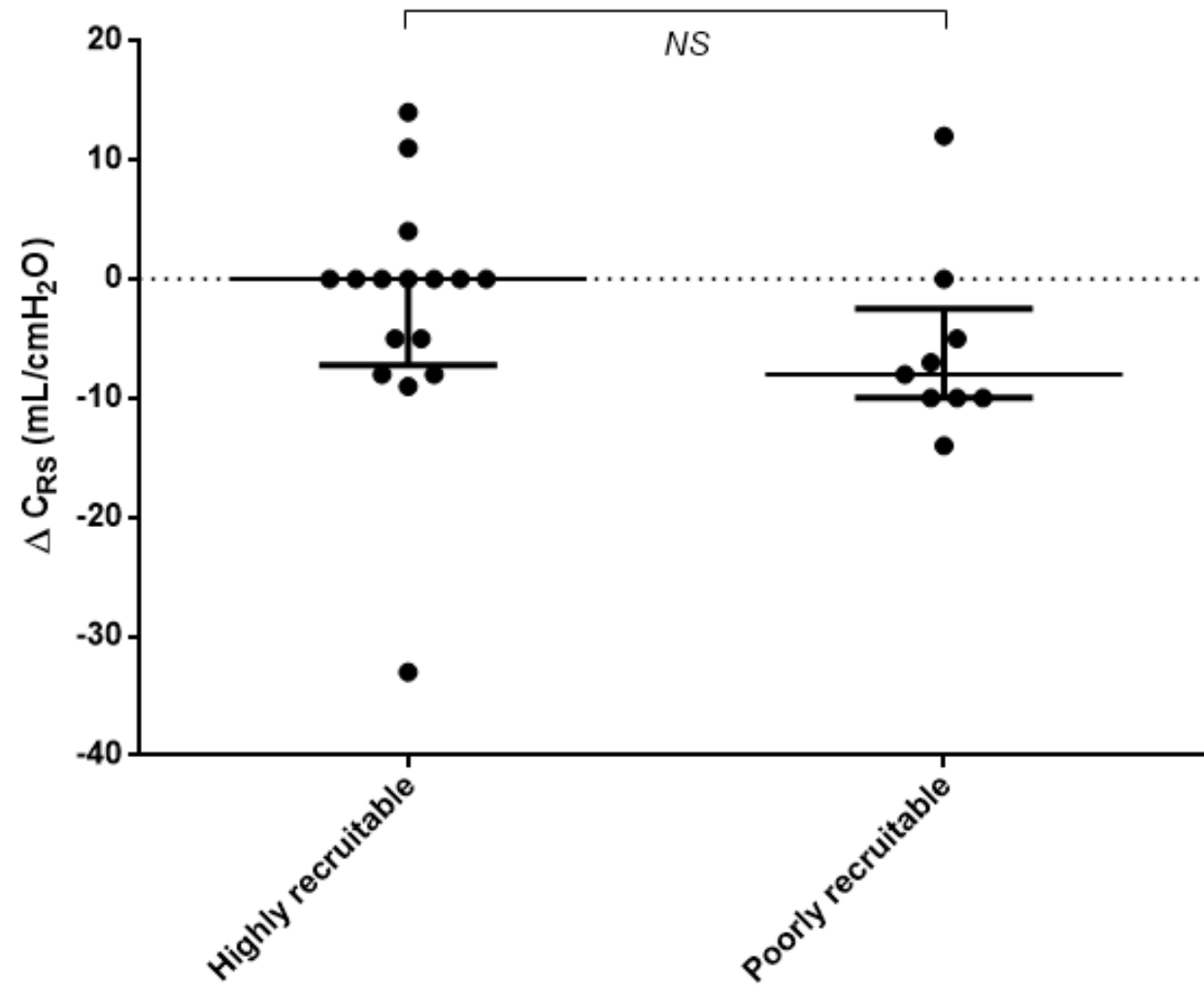

Supplement: Supplementary file 3 — Additional file 3: Figure S3. Distribution of changes in respiratory system compliance (∆CRS) from positive end-expiratory pressure (PEEP) 5 cmH2O to PEEP 15 cmH2O within 36 h after intubation in the highly recruitable and poorly recruitable patients groups. Horizontal lines represent median and interquartile range values. NS, not significant (p > 0.05). [file 13613_2020_675_MOESM3_ESM.pdf]

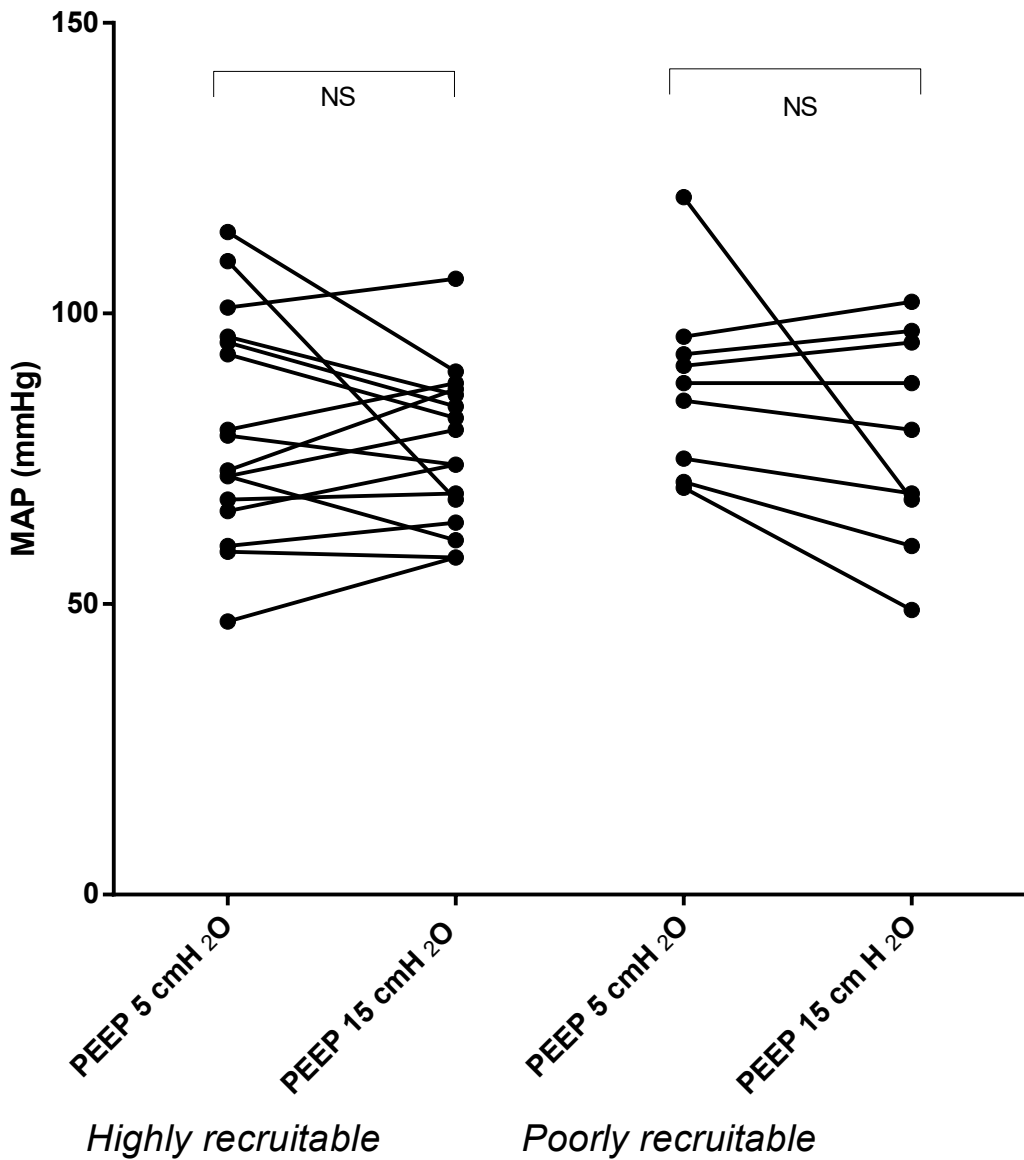

Supplement: Supplementary file 4 — Additional file 4: Figure S4. Distribution of mean arterial pressure (MAP) (A) and heart rate (B) at positive end-expiratory pressure (PEEP) 5 cmH2O and 15 cmH2O in the highly recruitable and poorly recruitable patients groups. NS, not significant (p > 0.05). [file 13613_2020_675_MOESM4_ESM.zip › 4a.pdf]

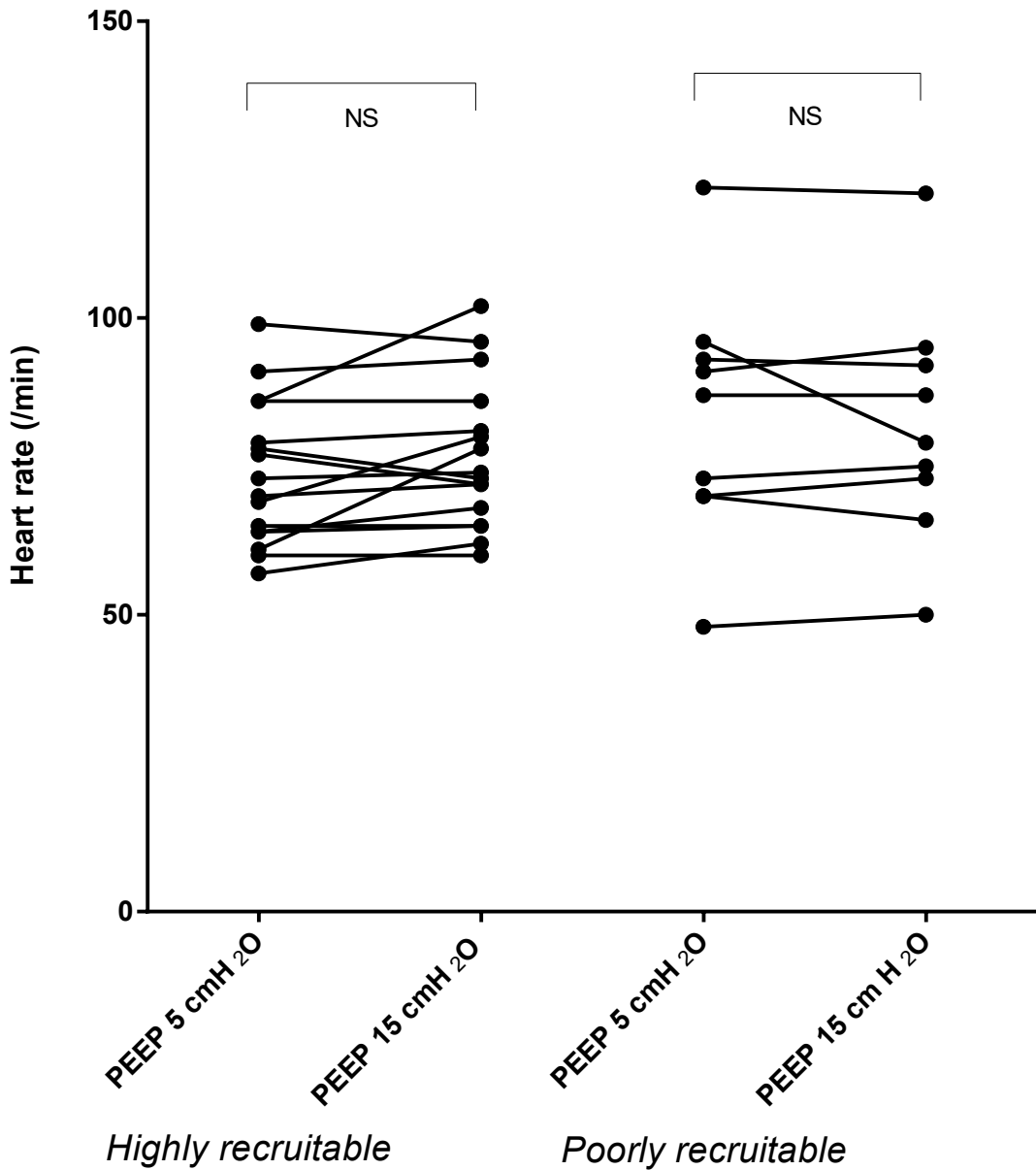

Supplement: Supplementary file 4 — Additional file 4: Figure S4. Distribution of mean arterial pressure (MAP) (A) and heart rate (B) at positive end-expiratory pressure (PEEP) 5 cmH2O and 15 cmH2O in the highly recruitable and poorly recruitable patients groups. NS, not significant (p > 0.05). [file 13613_2020_675_MOESM4_ESM.zip › 4b.pdf]

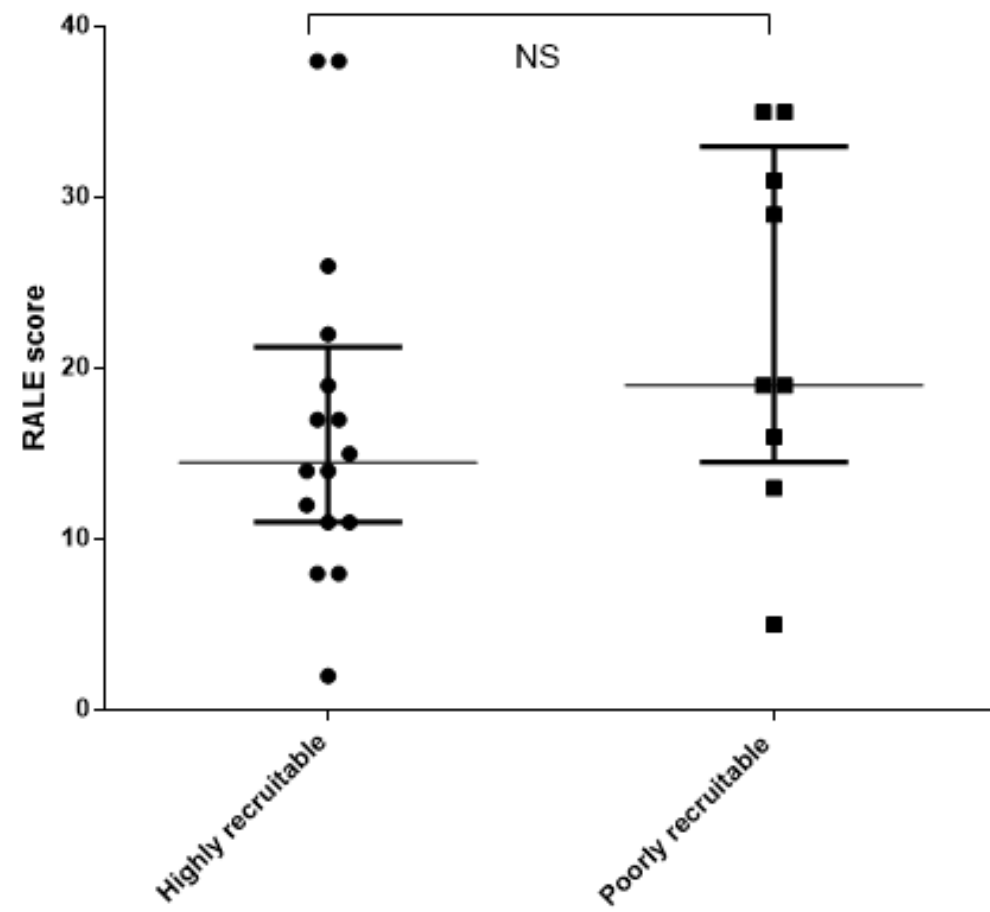

Supplement: Supplementary file 5 — Additional file 5: Figure S5. Distribution of the Radiographic Assessment of the quantity of Lung Edema (RALE) score at the day of intubation in the highly recruitable and poorly recruitable groups. NS, no significant (p > 0.05). Horizontal lines represent median and interquartile range values. [file 13613_2020_675_MOESM5_ESM.pdf]
